# Gait Training with a Dislocated Hip Spacer: A Case Study and Literature Review

**DOI:** 10.3390/jcm14155316

**Published:** 2025-07-28

**Authors:** Stefano Salvaderi, Valentina Liquori, Giovanni Zatti, Giorgio Ferriero, Francesco Negrini, Calogero Malfitano, Ludovit Salgovic, Paola Emilia Ferrara

**Affiliations:** 1 Physical and Rehabilitation Medicine Unit, Scientific Institute of Lissone, Istituti Clinici Scientifici Maugeri, 20851 Lissone, Italy; 2 Physical and Rehabilitation Medicine Unit, Scientific Institute of Tradate, Istituti Clinici Scientifici Maugeri IRCCS, 21049 Tradate, Italy; 3 Orthopaedic Department, San Gerardo Hospital, University of Milano-Bicocca, 20900 Monza, Italy; giovanni.zatti@unimib.it; 4 Department of Medicine and Surgery, University of Milano-Bicocca, 20900 Monza, Italy; 5 Department of Biotechnology and Life Sciences, University of Insubria, 21100 Varese, Italy; 6 Department of Biomedical Sciences for Health, University “La Statale”, 20133 Milan, Italy; calogero.malfitano@unimi.it; 7 Azienda di Servizi alla Persona Istituti Milanesi Martinitt e Stelline e Pio Albergo Trivulzio, 20146 Milan, Italy; 8 Department of Clinical Disciplines, Institute of Physiotherapy, Balneology and Medical Rehabilitation, University of Ss. Cyril and Methodius, 91701 Trnava, Slovakia; 9 Department of Geriatrics, Orthopedics and Rheumatology, Fondazione Policlinico Universitario “Agostino Gemelli” IRCCS, 00168 Rome, Italy; paolaemilia.ferrara@policlinicogemelli.it

**Keywords:** rehabilitation, physical therapy, hip arthroplasty, hip replacement, prosthesis-related infections, case report

## Abstract

**Background/Objectives:** Spacer dislocation is among the most frequent mechanical complications after revision total hip arthroplasty for periprosthetic hip infection. Spacer dislocations may be managed conservatively, but there are no guidelines on the rehabilitation of these patients, and the restriction of weight bearing is still under debate. **Methods:** We first report the case of a patient with hip spacer cranial dislocation, judged unfit to be surgically treated once more for a medium period, who started a rehabilitation program with partial weight bearing. **Results:** After two weeks of inpatient rehabilitation, the patient started to maintain the standing position with partial weight bearing on the affected side. Following hospital discharge we continued rehabilitation in the outpatient clinic. Despite the finding of the denervation of the ipsilateral quadriceps, three months after admission, she was able to walk for short distances using a walker, initially with the help of a therapist and then with supervision. About one year later, she was able to undergo the reimplantation of the definitive prosthesis. **Conclusions:** Despite the spacer dislocation, walking short distances is a feasible goal, even with assistance, wearing a brace and using a walker. Future research is needed to confirm and expand upon this observation and to understand the mechanisms underlying the development of neurological complications to implement effective prevention strategies.

## 1. Introduction

Infection is the third most common cause of revision total hip arthroplasty and the most frequent reason for the removal of the prosthesis [1]. Before the reimplantation of a definitive prosthesis (two-stage revision), a temporary antibiotic-loaded articulating spacer can be implanted to deliver antibiotics locally, to allow for weight bearing and to counter contracture formation [2].

The articulating spacer enables an early rehabilitation program first consisting of joint mobilization and then ambulation training with partial weight bearing [2,3]. However, this procedure is not free from complications, including mechanical failure, periprosthetic fractures, and spacer dislocations, which are the most common [4]. When the dislocated spacer is left in, in addition to hip pain, a sciatic nerve palsy may occur [5].

Another condition in which hip anatomy is lost is Girdlestone resection arthroplasty (GRA), a viable treatment option following failed total hip replacements. GRA is a surgical procedure that involves removing the femoral head and neck without replacement, leaving a crude articulation between the acetabulum and the proximal femur. It is often referred to as a salvage procedure, typically reserved for patients with significant medical or functional impairments who are at high surgical risk. Common outcomes after GRA include limb shortening, persistent pain, and reduced mobility and independence [6]. Although rare, femoral nerve palsy may occur following the procedure [7]. While GRA may allow patients to walk immediately postoperatively [8], they typically become dependent on walking aids [6].

Although there is extensive literature on postoperative management and walking ability in patients who have undergone GRA [6], no guidelines currently exist for patients with hip spacer dislocation. The restriction of weight bearing in these cases remains a topic of debate. To date, there are no reports describing the functional outcomes of patients with a dislocated hip spacer who are permitted to undergo walking training.

Therefore, we report first the case of a patient with spacer cranial dislocation who was treated conservatively and underwent a rehabilitation program with partial weight bearing.

## 2. Materials and Methods

**Figure 1:**
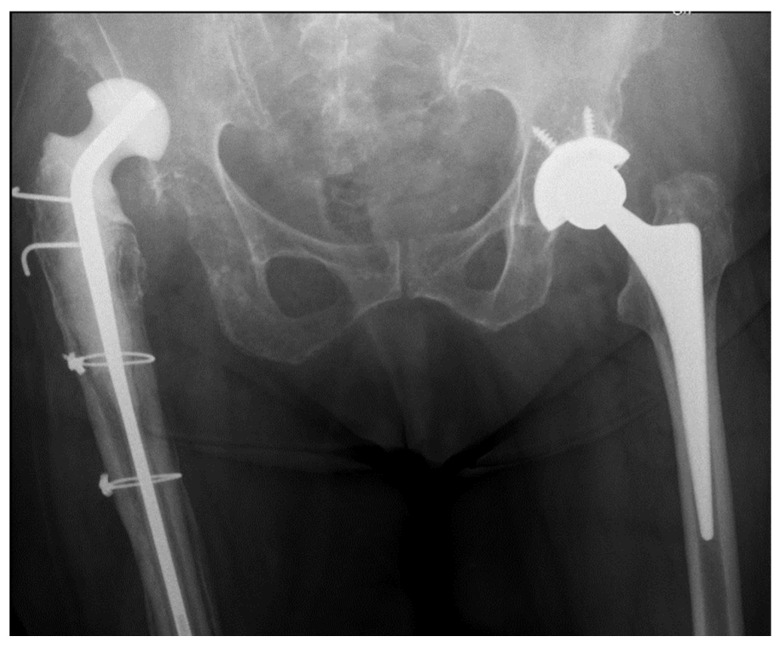
Spacer dislocation at the right hip.

In summer 2019, an 81 yr old right-hand-dominant woman came to us for observation, transferred after status epilepticus, in acute kidney failure, and with a dislocated spacer at the right hip (Figure 1).

In 2009, she underwent elective right hip replacement for osteoarthritis, complicated after a few months by joint dislocation, requiring acetabular cup revision surgery. In 2015, she underwent elective replacement even at the left hip. After a long period of well-being, in 2016, she presented a periprosthetic right femur fracture treated by second revision surgery and cerclage wires. One month later, during inpatient rehabilitation, another dislocation with a new femur fracture occurred, treated by third revision surgery. A bacterial wound culture determined the presence of bacteria. Two days after the culture result, she underwent another revision surgery. An analysis of intraoperative samples determined the presence of infection at the site of total hip arthroplasty. She started antimicrobial treatment and resumed the rehabilitation program, initially with touch weight bearing to increase progressively to walking with partial weight bearing.

She continued to feel right leg and back pain and experience difficulty in walking, despite receiving several long-lasting rehabilitation treatments as an outpatient. To diagnose this pain, at the end of 2018, she underwent magnetic resonance imaging for the lumbar spine. This exam showed the presence of a fragment of disk material compressing the right root in L4–L5. The orthopedist found a weakness in her right leg and asked for new blood exams and hip magnetic resonance imaging (MRI). The results were suggestive for prosthetic joint infection. The infectious diseases specialist prescribed an 18 F-FDG-PET. The result was again positive but not confirmed by a synovial fluid joint aspiration. Since pain and difficulty in walking were present, and radiological imaging confirmed infection, it was decided to plan a two-stage revision using an articulating antibiotic-loaded spacer. In spring 2019, she underwent the first-stage procedure consisting of removing the infected prosthesis, surgical debridement, the implantation of the spacer, and long-term antibiotic therapy. Then, she was admitted to a Physical and Rehabilitation Medicine (PRM) department at another hospital. After a few weeks of inpatient stay, while lying in bed, she felt sudden pain and lost functionality in her right hip. Imaging showed a cranial dislocation of the spacer (Figure 1). An attempt at reduction was unsuccessful. Since it was decided to continue treatment conservatively, she was admitted again to the previous PRM department. Unfortunately, drug overuse caused an acute kidney failure, with consequent admission to the acute department, and then a metabolic encephalopathy, resulting in status epilepticus.

## 3. Results

Once status epilepticus resolved, the orthopedic surgeon prescribed her gait training with progressive partial weight bearing and the use of an extra lift for the heel height of the right shoe. However, pain, general clinical condition, and the concomitant weakness of both legs, mainly at the right quadriceps femoris, did not allow her to perform any weight bearing. At that time the orthopedic surgeon’s team judged the patient unfit to be treated surgically once more, so she was transferred to our rehabilitation department.

At admission at our department, the clinical examination showed the following results: The strength of the right quadriceps femoris and hip abductors was very poor, rated, respectively, at 1/5 and 2/5 on the Medical Research Council (MRC) scale. No sensory deficits. Postural changes were possible with the assistance of two operators. Standing position, balance, and walking were not feasible.

She started with a rehabilitation program that consisted of more than one hour of exercises, seven days per week. The program consisted of the following: joint mobilization and strengthening exercises for four limbs, focused on the right hip, and training for postural changes and trunk control in the sitting and upright positions, using a standing frame and an electronic tilt table, without weight bearing and, two weeks after admission, with partial weight bearing at the right side, according to pain intensity. The extra lift added to the heel height of the right shoe was 1.5 cm. For pain therapy, she was treated with gabapentin and electrotherapy (Table 1).

After two weeks, she started to maintain the standing position with partial weight bearing on the right side. An X-ray performed one week after the partial weight bearing confirmed that the hip situation was unchanged. On the following visit, the orthopedic surgeon prescribed gait training with an axillary walker and a locked knee brace. After one month, she was discharged to continue her rehabilitation program at our outpatient clinic, three times per week, 90′ per session. Clinically, the active extension of the right knee was abolished, but she had no pain (Numerical Rating Scale, NRS = 0/10). She was able to stand up with double support, but walking was not possible (Functional Independence Measure, FIM = 56/126).

Electromyography showed a total denervation of the quadriceps, severe denervation with collateral innervation at the psoas and great adductor, and the reinnervation of the tibialis anterior muscles on the right side. The conclusion was a possible severe lumbar radiculopathy (L2–L3 and, less evident, L4) to be further investigated with an MRI. The MRI confirmed multiple lumbar radiculopathy several radicular compressions and, more relevantly, revealed the presence of fluid collection in the right iliac fossa (a previous computerized tomography (CT) scan—performed at the end of May when she was admitted to the intensive care unit—already showed an intramuscular hemorrhage (15.6 × 12.1 × 15.8 cm) at the right psoas muscle, treated with embolization).

About one month after inpatient discharge, she was able to walk for short distances using a walker, initially with the help of a therapist and then with supervision (Table 2).

At the end of 2019, the orthopedic surgeon planned the removal of the spacer, but, unfortunately, after 10 days, she presented again with the dislocation of the left hip prosthesis (the opposite side of the dislocated spacer), treated with closed reduction. Once weight bearing was allowed, she started to walk again using a walker with partial weight bearing. In February 2020, the outpatient rehabilitation was discontinued with the maximal progress identified.

After one year the patient was still able to walk for short distances—achieving a sufficient quadriceps strength recovery—using the walker with acceptable pain. There were no symptoms or signs of infection. In November 2020, she was able to undergo the reimplantation of the definitive prosthesis. As of 2025 she is able to walk for medium distances using a crutch.

## 4. Discussion

Patients with dislocations often present a poor clinical outcome [9]. The rehabilitation of patients with permanent hip spacer dislocation is a real challenge for any clinician in the absence of guidelines, standardized protocols, or narrative reports. Often these patients—despite rehabilitation—may even present with chronic disuse muscle atrophy after the previous total hip arthroplasty, complicated by a recent hospitalization [10]. Low skeletal muscle mass and muscle weakness may be further exacerbated by sarcopenia in these patients, who are usually old and frail [11].

After spacer dislocation, several approaches have been reported, and one is the conservative treatment [5], with an unclear functional outcome. In 2010, a prospective comparative study described 18 patients with spacer cranial dislocation [12]. These patients were not allowed to bear weight on the affected leg. Similarly, Pattyn et al. reported that 10 out of 61 patients treated by two-stage revision had spacer dislocation, and 8 of them were treated conservatively with restricted weight bearing [13]. They concluded that most dislocations can be managed conservatively, but it was not reported whether the restriction of weight bearing should be permanent. Erivan et al. [14] reported on one patient who received the indication to keep their dislocated spacer until his death, without any mention of weight bearing. In 2014, Bori et al. [5] described their experience with 74 patients after the two-stage revision. Spacer dislocation occurred in seven of them. None were discharged with the spacer dislocation, but most of them were not able to undergo a subsequent implant procedure for a definitive arthroplasty. Bori et al. concluded that in the future the dislocation would not be reduced, and patients should perform less load bearing on the affected limb [5]. Recently, the same research team reported on their experience with another 6 patients who had spacer dislocation from a sample of 67 patients [9]. Only one patient was deemed to be medically unfit to be reoperated on and had to retain the dislocated spacer. Unfortunately, whether they were allowed to walk with crutches in order to keep weight bearing to a minimum was not reported, although the same authors stated that this procedure was successfully implemented at their hospital.

While joint mobilization is considered essential to prevent the stiffness of muscular and articular tissues, gait training may be considered a risky activity and weight bearing an impossible goal. However, walking with need of a frame support is possible in patients with hip resection arthroplasty, a salvage procedure consisting of the removal of the hip prosthesis without a prosthetic replacement [15]. In addition, it is well known that early weight bearing improves the quality of life and functionality [16]. On the other hand, the functional outcome after hip resection arthroplasty is severely impaired due to hip instability, pseudo-arthrosis, and a high level of pain at rest and during activity [15].

Unfortunately, our patient was judged unfit to be surgically treated once more, for a moderate period of time (up to one year). To increase her quality of life, it was decided to include in her rehabilitation program gait training with progressive partial weight bearing, according to pain intensity. She started with exercises to control weight shift in the standing position and partial weight bearing, first using an electronic tilt table and then a standing frame. The diagnosis of the denervation of the right lower limb’s muscles required the orthotic management of the weak leg, complicating the patient’s compliance in walking daily in her own home. Wearing the leg brace and using a walker, the patient was able to rise from a chair and walk a few meters, initially requiring assistance.

The denervation of the lower limb’s muscles has rarely been reported in patients with hip spacer dislocation. Garceau et al. reported in their retrospective study that only 1 patient (out of 24) with spacer dislocation developed sciatic nerve palsy after final hip prosthesis insertion [2]. Pattyn et al. described another patient (out of 10) who had sciatic nerve palsy treated by the intervention of open reduction [13]. For our patient, at the beginning, it was unclear whether the spacer dislocation—eventually together with weight bearing—was the direct cause of such neuropathy. In fact, she was affected by chronic right sciatic pain in the well-known compression of the right root in the L4–L5 spine, caused by a fragment of disk material.

A potential cause of femoral nerve palsy may be the altered anatomical course of the femoral nerve into the quadriceps muscle group, as a result of spacer migration. A recent cadaveric study reported that GRA (a comparable anatomical condition) can lead to femoral nerve displacement. In particular, stress was observed at the lateral oblique division of the femoral nerve, where the major psoas muscle acts as a hindrance. Such stress may contribute to the development of femoral nerve palsy [6].

Femoral nerve palsy is also recognized as an uncommon complication following two-stage revision arthroplasty [17]. However, in the present case, the presence of marked weakness in hip flexion and knee extension—confirmed by neurophysiological assessment—suggests, as an alternative etiology, an L2–L3 radiculopathy secondary to a spontaneous psoas hematoma. This condition is well documented in the literature as a common consequence of psoas hematoma, particularly in patients who stay in intensive care units [18].

## 5. Conclusions

In conclusion, gait training following a permanent dislocated hip spacer presents a complex clinical challenge that requires a nuanced understanding of biomechanics, tissue healing, and patient-specific factors. The primary goal of rehabilitation in this context is to restore functional mobility while minimizing mechanical stress on the affected hip and surrounding tissues. Key interventions include range-of-motion exercises, muscle strengthening, and progressive weight-bearing activities, all of which must be individualized based on the patient’s functional status, comorbidities, and tolerance.

Our patient was able to walk for short distances, wearing a brace and using a walker, presenting a feasible goal. This result was maintained after one year. Future research is needed to confirm and inform this observation and to understand the mechanisms underlying the development of neurological complications associated with a hip spacer implant and impacts on the femoral and sciatic nerves, in order to implement any possible effective management strategy.

## Figures and Tables

**Table 1:** Phases of rehabilitation program.

Admission	After 2 Weeks	After One Month, at Start of Day at Hospital
ROM and stretching exercises for right lower limb	ROM and stretching exercises for right lower limb	ROM and stretching exercises for right lower limb
Open and closed chain strengthening exercises for four limbs	Open and closed chain strengthening exercises for four limbs	Open and closed chain strengthening exercises for four limbs
Training for postural changes	Training for postural changes	Training for postural changes
Trunk control training in sitting and upright positions without weight bearing on right lower limb	Trunk control training in sitting and upright positions with partial weight bearing on right lower limb	Trunk control training in sitting and upright positions with partial weight bearing on right lower limb
Transcutaneous electrical nerve stimulation for pain relief	Transcutaneous electrical nerve stimulation for pain relief	Gait training with partial weight bearing on right lower limb

**Table 2:** The spatial gait parameters of the patient walking with a two-wheel walker and wearing a knee brace, measured by a pressure-sensitive walkway 7 months after the spacer dislocation.

Gait Parameter	Value
Steps	17
Distance (cm)	392.53
Time (s)	20.05
Speed (cm/s)	19.6
Cadence (steps/min)	50.9
Right step length (cm)	35.88
Left step length (cm)	11.72
Right step time (s)	1.52
Left step time (s)	0.87
Right step width (cm)	15.33
Left step width (cm)	15.37

## Data Availability

The original contributions presented in the study are included in the article, further inquiries can be directed to the corresponding authors.

## Abbreviations

The following abbreviations are used in this manuscript:

MRI	Magnetic resonance imaging
F-FDG-PET	Fluorodeoxyglucose positron emission tomography
PMR	Physical and rehabilitation medicine
FIM	Functional Independence Measure
NRS	Numeric rating scale
CT	Computed tomography
